# Associations between back pain across adulthood and spine shape in early old age in a British birth cohort

**DOI:** 10.1038/s41598-018-34628-9

**Published:** 2018-11-05

**Authors:** Stella G. Muthuri, Anastasia V. Pavlova, Fiona R. Saunders, Rebecca J. Hardy, Jennifer S. Gregory, Rebecca J. Barr, Kathryn R. Martin, Judith E. Adams, Diana Kuh, Richard M. Aspden, Rachel Cooper

**Affiliations:** 1 MRC Unit for Lifelong Health and Ageing at UCL, 33 Bedford Place, London, WC1B 5JU UK; 2 Arthritis and Musculoskeletal Medicine, School of Medicine, Medical Sciences and Nutrition, University of Aberdeen, Aberdeen, AB25 2ZD UK; 3 Medicines Monitoring (MEMO) Research, Division of Molecular & Clinical Medicine, School of Medicine, University of Dundee, Mailbox 2 Level 7, Ninewells Hospital & Medical School, Dundee, DD1 9SY UK; 4 Manchester Academic Health Science Centre, Central Manchester University Hospitals NHS Foundation Trust, Manchester Royal Infirmary, Oxford Road, Manchester, M13 9WL UK

## Abstract

We aimed to examine whether back pain across adulthood was associated with spine shape at age 60–64 years. Data were from 1405 participants in the MRC National Survey of Health and Development, a nationally representative British birth cohort. Back pain was ascertained during nurse interviews at ages 36, 43, 53 and 60–64 years. Cumulative exposure to back pain was then derived by counting the number of ages at which back pain was reported. Statistical shape modelling was used to characterise thoracolumbar spine shape using lateral dual-energy x-ray absorptiometry images which were ascertained at age 60–64 years. Linear regression models were used to test associations of spine shape modes (SM) with: (1) cumulative exposure to back pain; (2) back pain reports during different periods of adulthood. After adjusting for sex, higher cumulative exposure to back pain across adulthood was associated with wedge-shaped L4-5 disc (lower SM4 scores) and smaller disc spaces (higher SM8 scores) in both sexes. In addition, reporting of back pain at ages 53 and/or 60–64 years was associated with smaller L4-5 disc space (lower SM6 scores) in men but not women. These findings suggest that back pain across adulthood may be associated with specific variations in spine shapes in early old age.

## Introduction

Back pain is the most prevalent musculoskeletal condition worldwide and a leading cause of years lived with disability^1^. It affects people of all ages^2,3^ and its consequences for both the individual and society are considerable^2,4–7^. Back pain is a complex multi-factorial disorder; biomechanical, biological, psychological and social factors play a role in its onset, persistence and severity^8^. Furthermore, there have been suggestions that variations in the anatomical shape of the spine may predispose some individuals to increased risk of back pain and spinal injury^9–11^.

Within a population, spine shape varies with some individuals having curvier (lordotic) spines than others^12,13^. Recent studies have shown that each individual has an intrinsic spine shape that is partly maintained during postural changes^10,11^. This influences the preferred manual lifting technique used^10,11^ raising the possibility that spinal shape may play a role in the susceptibility to and recovery from low back pain and may predispose individuals with certain variations in shape to increased risk of spinal injury.

Spine morphology is also likely to reflect age-related degenerative changes which may result in subsequent adverse health outcomes including back pain^14–16^. Similarly, pre-existing degenerative back disorders including spinal osteoarthritis, lumbar disc degeneration, osteoporosis and vertebral fractures are often accompanied by structural changes in the spinal shape as a consequence of disease processes and have been linked to back pain and reduced spine flexibility and mobility^17,18^. Other studies suggest that persistent back pain or trauma may be associated with decreased mobility and flexibility of muscles and joints^9,19^.

To date most epidemiological studies of the relationship between back pain and spine shape have examined radiological abnormalities^20,21^ or sagittal spinal curves^22,23^. However, these studies face important limitations that lead to uncertainty about the relationship between back pain and intrinsic spine morphology in the general population^22,23^. For example, many studies are cross-sectional, and/or have assessed pain at a single prior age thereby failing to acknowledge that back pain is often a recurrent, intermittent problem with prior history predictive of future episodes and have not accounted for possible confounders^2,24^. They have also mainly used geometric features such as segmental angles to characterise spinal curvature which may not capture systematic variations in spine morphology^12,13,22^.

Using data from a large, nationally representative sample, we aimed to address these gaps in the literature by examining whether cumulative exposure to back pain across adulthood is associated with spine shape at age 60–64 years. As different profiles of back pain across adulthood have been identified^25^ we also aimed to examine whether back pain reports during different periods of adulthood were associated with spine shape.

## Results

**Figure 1:**
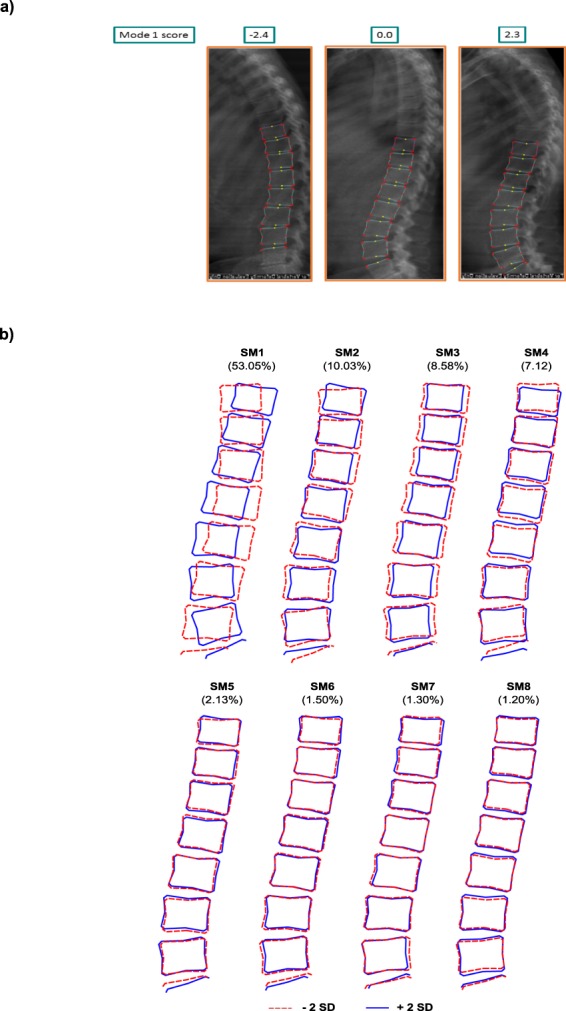
(**a**) Shows representative DXA images of the NSHD cohort sorted by their spine mode (SM) 1 score, with the template used to mark the anatomical landmarks of the vertebral bodies overlaid onto the image and (**b**) illustrates the variation in lumbar spine shape and the percentage variance detected by spine modes (SM) 1 to 8 shown as ± 2 SD from the mean of zero for the whole cohort.

Women had higher mean scores for spine mode (SM) 1, 3 and 8 than men but lower scores for SM6. Figure 1(a) shows representative DXA images with the template used to highlight the regions of the spine included in the shape model of SM1 and Fig. 1(b) shows illustrations of each SM and descriptions of key features identified by these modes are summarised in Table 1.

**Table 1:** Characteristics of spine modes (SM) 1 to 8 in the MRC National Survey of Health and Development.

SM	Lower values	Higher values
1	• flatter or less lordotic lumbar curve with slight thoracic kyphosis	• greater lumbar lordosis
2	• uneven distribution, more S-shaped or snaking	• evenly distributed curvature
3	• larger relative anterior-posterior (a-p diameter) vertebral dimensions	• smaller relative a-p diameter vertebral dimensions
4	• anterior disc wedging caudad with a compensatory posterior vertebral wedging cephalad	• relatively uniform disc and vertebral heights, leading to a straighter spine
5	• Thoracic section tending towards a kyphosis but a flatter lordosis (anti-clockwise rotation at T10 and L3, clockwise rotation at L5). • Smaller L5 anterior tilt and narrower L4/L5 disc space.	• Straighter T10-L2 section with a slightly greater lordosis from L3-L5 (clockwise rotation at T10 and L3, anti-clockwise rotation at L5). • Greater L5 anterior tilt and wider L4/L5 disc space.
6	• Smaller relative a-p diameters cranially; smaller than average at T10, T11 but wider than average at L3-L5. • Smaller L4/L5 disc space.	• More uniform relative a-p diameters; greater than average at T10, T11 but smaller than average at L3-L5. • Greater L4/L5 disc space.
7	• Smaller T10-T12, but larger than average L4-L5 relative a-p diameter. Squarer vertebral bodies in thoracic section.	• Greater T10-T12 but smaller than average relative L4-L5 a-p diameter.
8	• Smaller vertebral body heights, relatively larger disc spaces.	• Taller vertebral body heights, relatively smaller disc spaces.

SM: spine mode.

Table 2 presents the characteristics of the 1405 study participants for whom back pain data were available on at least 3 visits.

**Table 2:** Characteristics of the MRC National Survey of Health and Development study sample^†^ at age 60–64 stratified by sex.

	Male	Female	p-value^‡^
Sex, N (%)	676 (48.1)	729 (51.9)	
Age at nurse visit (years); mean (SD), n = 1404	63.2 (1.18)	63.3 (1.09)	0.13
**Spine modes; mean (SD), n** = **1, 405**			
SM1	−0.07 (0.96)	0.06 (1.02)	0.014
SM2	0.01 (1.01)	−0.02 (0.98)	0.60
SM3	−0.51 (0.98)	0.46 (0.77)	<0.001
SM4	0.04 (0.98)	−0.06 (1.02)	0.07
SM5	0.03 (1.00)	−0.03 (1.00)	0.24
SM6	0.21 (0.98)	−0.18 (1.00)	<0.001
SM7	0.04 (1.03)	−0.02 (0.97)	0.24
SM8	−0.24 (0.99)	0.24 (0.93)	<0.001
**Back pain, n (%)**			
Age 36 years; n = 1, 323			0.11
No	533 (84.2)	558 (80.9)	
Yes	100 (15.8)	132 (19.1)	
Age 43 years; n = 1, 369			0.76
No	492 (74.7)	525 (73.9)	
Yes	167 (25.3)	185 (26.1)	
Age 53 years; n = 1, 362			0.60
No	448 (69.6)	490 (68.3)	
Yes	196 (30.4)	228 (31.8)	
Age 60–64 years; n = 1, 401			0.73
No	490 (72.6)	521 (71.8)	
Yes	185 (27.4)	205 (28.2)	
Cumulative back pain score, n = 1405			0.48
0 (no back pain)	318 (47.0)	320 (43.9)	
1	177 (26.2)	187 (25.7)	
2	100 (14.8)	125 (17.2)	
3–4 back pain reports	81 (12.0)	97 (13.3)	
Timing of back pain^¶^, n = 1405			0.55
No back pain	318 (47.0)	320 (43.9)	
Mid-adulthood only (36/43 years)	69 (10.2)	81 (11.1)	
Later adulthood only (53/60–64 years)	161 (23.8)	172 (23.6)	
Persistent back pain (mid and later adulthood)	128 (18.9)	156 (21.4)	
**Covariates**			
BMI (kg/m^2^) at age 60–64 years; mean (SD), n = 1405	27.7 (3.84)	27.3 (4.61)	0.13
Height (cm) at age 60–64 years; mean (SD), n = 1405	175.2 (6.35)	162.2 (5.76)	<0.001
Spine aBMD (g/cm^2^) at age 60–64; mean (SD), N = 1405	1.05 (0.18)	0.94 (0.16)	<0.001
Occupational class at age 53 years, n = 1405			<0.001
High (I/II)	413 (61.1)	335 (46.0)	
Middle (IIINM/IIIM)	213(31.5)	301 (41.3)	
Low (IV/V)	50 (7.4)	93 (12.8)	
Participation in sports, vigorous leisure activities or exercise at age 60–64 years, (n = 1405)			0.26
Regularly active (≥5 times/month)	167 (24.7)	192 (26.3)	
Moderately active (1–4 times/month)	101 (14.9)	127 (17.4)	
Inactive	408 (60.4)	410 (56.2)	
Symptoms of anxiety and depression (GHQ-28) at age 60–64 years (n = 1405)			<0.001
No (≤4)	595 (88.0)	579 (79.4)	
Yes (>4)	81 (12.0)	150 (20.6)	
Number of children, n = 1405			0.12
None	87 (12.9)	82 (11.3)	
1	67 (9.9)	87 (11.9)	
2	344 (50.9)	337 (46.2)	
3 or more	178 (26.3)	223 (30.6)	

BMI: Body mass index; GHQ: general health questionnaire; SD: standard deviation; SM: spine mode; aBMD: areal bone mineral density.

^†^Sample restricted to those participants with back pain data on at least 3 data waves.

^‡^Comparison of sexes using student t-test or chi-square tests, as appropriate.

^¶^Mid-adulthood only: back pain reported at age 36 and/or 43 years only; later adulthood only: back reported at age 53 and/or 60–64 years only; Persistent: Back reported in both mid and later adulthood.

In both sexes, the prevalence of back pain increased until age 53 years and no sex differences were observed in the cumulative exposure or timing of back pain. In terms of covariates, men were taller, more likely to have a higher occupational class and less likely to report symptoms of anxiety and depression than women (Table 2). Women were more likely to have a lower spine areal BMD than men (Table 2).

Table 3 shows associations between cumulative reports of back pain across adulthood and each spine mode. Compared to those who did not report back pain, those who had greater cumulative exposure to back pain across adulthood were more likely to have a larger relative anterior-posterior (a-p) diameter (lower SM3 scores), wedge-shaped L4-5 disc (lower SM4 scores) and smaller disc spaces (higher SM8 scores), after adjusting for sex. In men, there was evidence that the association between cumulative reports of back pain and SM6 was non-linear so that back pain reported at one or two ages in adulthood was associated with smaller L4-5 disc space (lower SM6 scores), (p for sex interaction = 0.048). Most of these associations, with the exception of SM3 and SM4, remained after adjusting for potential confounders.

**Table 3:** Associations between cumulative back pain score and spine modes (N = 1405).

Number of ages back pain was reported	Sex adjusted model^†^		Fully-adjusted model^¶,†^	
	β (95%CI)	P trend^‡^	β (95%CI)	P trend^‡^
**SM1**				
0	0	0.995	0	0.95
1	−0.06 (−0.18, 0.07)		−0.06 (−0.18, 0.07)	
2	−0.01 (−0.16, 0.14)		−0.02 (−0.17, 0.14)	
3–4	0.01 (−0.15, 0.17)		0.01 (−0.16, 0.17)	
**SM2**				
0	0	0.20	0	0.17
1	0.01 (−0.12, 0.14)		0.02 (−0.11, 0.15)	
2	0.04 (−0.11, 0.19)		0.05 (−0.11, 0.2)	
3–4	0.11 (−0.06, 0.28)		0.12 (−0.04, 0.29)	
**SM3**				
0	0	**0.021**	0	0.28
1	−0.1 (−0.21, 0.02)		−0.08 (−0.19, 0.03)	
2	−0.13 (−0.27, 0.001)		−0.08 (−0.21, 0.06)	
3–4	−0.13 (−0.28, 0.01)		−0.05 (−0.20, 0.09)	
**SM4**				
0	0	0.055	0	0.14
1	−0.04 (−0.17, 0.09)		−0.04 (−0.17, 0.09)	
2	0.002 (−0.15, 0.15)		0.02 (−0.13, 0.18)	
**3–4**	**−0.21 (−0.38, −0.04)**		**−0.18 (−0.34, −0.01)**	
**SM5**				
0	0	0.089	0	0.13
1	−0.05 (−0.18, 0.08)		−0.05 (−0.18, 0.08)	
2	0.03 (−0.12, 0.18)		0.04 (−0.11, 0.19)	
**3–4**	**−0.20 (−0.37, −0.04)**		**−0.19 (−0.36, −0.02)**	
**SM6** ^†^				
Men, n = 676				
0	0	0.041^‡^	0	0.04^‡^
1	−0.21 (−0.39, −0.03)		−0.19 (−0.38, −0.01)	
2	−0.33 (−0.55, −0.11)		−0.29 (−0.51, −0.07)	
3–4	−0.10 (−0.34, 0.14)		−0.05 (−0.29, 0.20)	
Women, n = 729				
0		0.09		0.12
1	0.06 (−0.12, 0.24)		0.05 (−0.13, 0.23)	
2	−0.08 (−0.28, 0.13)		−0.08 (−0.29, 0.13)	
3–4	−0.20 (−0.43, 0.02)		−0.19 (−0.42, 0.04)	
**SM7**				
0	0	0.68	0	0.66
1	0.05 (−0.08, 0.18)		0.04 (−0.09, 0.17)	
2	−0.06 (−0.22, 0.09)		−0.07 (−0.22, 0.09)	
3–4	0.08 (−0.08, 0.25)		0.09 (−0.08, 0.26)	
**SM8**				
0	0	**<0.001**	0	**<0.001**
1	0.09 (−0.03, 0.22)		0.09 (−0.03, 0.21)	
2	0.25 (0.11, 0.40)		0.23 (0.08, 0.38)	
3–4	0.30 (0.14, 0.46)		0.27 (0.11, 0.43)	

SM: spine mode.

^†^Combined analyses presented except mode 6 which was sex-stratified, sex-interaction, p = 0.048 (unadjusted model); ^‡^p value for test of heterogeneity across groups when there was evidence of a deviation from a linear trend; ^¶^adjusted for sex, current BMI, spine aBMD, own occupational class, physical activity, GHQ-28 and number of children.

No associations were found between cumulative reports of back pain and modes which described total amount of curvature within L5 to T10 (SM1); differences in the distribution of curvature along the length of the spine (SM2); vertebral rotation at T10, L3 and L5 with resulting minor variations of lordosis and kyphosis and varying L4/L5 disc space (SM5); or minor variation in relative a-p diameter at T10-T12 and at L4-L5 (SM7).

When the associations of timing of back pain with spine shape were examined (Table 4), there was some evidence in both sexes that persistent back pain (i.e. reports of pain in both mid and later adulthood) were associated with lower SM3 (larger relative a-p vertebral diameter) and lower SM4 (wedge-shaped L4-5 disc) scores in sex-adjusted models. Men who reported back pain in later adulthood only (at ages 53 and/or 60–64 years) or reported persistent back pain also had lower SM6 scores (smaller L4-5 disc space scores) (sex interaction, p = 0.048). In addition, those who reported back pain in mid-adulthood only or reported persistent pain had higher mean SM8 scores (smaller disc spaces). After adjustment for covariates, back pain reports in mid and later adulthood remained associated with lower SM6 and higher SM8 scores, respectively; and persistent back pain remained associated with higher SM8 scores. However, associations with SM3 and SM4 were fully attenuated. No associations were observed between back pain reports during different age periods and SM1, 2, 5 or 7.

**Table 4:** Associations between timing of back pain and spine modes (N = 1405).

Timing of back pain*	Sex adjusted model^†^		Fully-adjusted model^¶,†^	
	β (95%CI)	P value	β (95%CI)	P value
**SM1**				
No back pain	0	0.28	0	0.28
Mid-adulthood only	0.09 (−0.09, 0.27)		0.09 (−0.09, 0.27)	
Later adulthood only	−0.09 (−0.22, 0.04)		−0.09 (−0.23, 0.04)	
Persistent	−0.01 (−0.15, 0.13)		−0.02 (−0.16, 0.12)	
**SM2**				
No back pain	0	0.35	0	0.34
Mid-adulthood only	−0.04 (−0.22, 0.13)		−0.03 (−0.21, 0.15)	
Later adulthood only	0.02 (−0.11, 0.15)		0.03 (−0.10, 0.16)	
Persistent	0.11 (−0.03, 0.25)		0.12 (−0.02, 0.26)	
**SM3**				
No back pain	0	0.089	0	0.40
Mid-adulthood only	−0.14 (−0.30, 0.01)		−0.12 (−0.27, 0.04)	
Later adulthood only	−0.09 (−0.21, 0.03)		−0.06 (−0.18, 0.05)	
Persistent	**−0.13 (−0.25, −0.01)**		−0.06 (−0.18, 0.06)	
**SM4**				
No back pain	0	0.22	0	0.49
Mid-adulthood only	−0.07 (−0.25, 0.11)		−0.07 (−0.24, 0.11)	
Later adulthood only	−0.003 (−0.14, 0.13)		−0.003 (−0.14, 0.13)	
Persistent	**−0.14 (−0.28, −0.0003)**		−0.10 (−0.24, 0.04)	
**SM5**				
No back pain	0	0.27	0	0.33
Mid-adulthood only	0.05 (−0.13, 0.23)		0.05 (−0.13, 0.23)	
Later adulthood only	−0.07 (−0.20, 0.06)		−0.07 (−0.21, 0.06)	
Persistent	−0.11 (−0.25, 0.03)		−0.10 (−0.24, 0.04)	
**SM6** ^†^				
*Men, n* = 676				
No back pain	0	**0.027**	0	0.067
Mid-adulthood only	−0.13 (−0.39, 0.12)		−0.12 (−0.38, 0.13)	
Later adulthood only	**−0.26 (−0.45, −0.08)**		**−0.24 (−0.43, −0.05)**	
Persistent	**−0.21 (−0.41, −0.01)**		−0.16 (−0.37, 0.04)	
*Women, n* = *729*				
No back pain	0	0.23	0	0.29
Mid-adulthood only	−0.15 (−0.39, 0.09)		−0.15 (−0.39, 0.09)	
Later adulthood only	0.07 (−0.12, 0.25)		0.06 (−0.13, 0.24)	
Persistent	−0.11 (−0.31, 0.08)		−0.11 (−0.30, 0.09)	
**SM7**				
No back pain	0	0.81	0	0.79
Mid-adulthood only	−0.03 (−0.21, 0.15)		−0.04 (−0.21, 0.14)	
Later adulthood only	0.02 (−0.11, 0.15)		0.02 (−0.12, 0.15)	
Persistent	0.06 (−0.08, 0.20)		0.06 (−0.08, 0.20)	
**SM8**				
No back pain	0	**<0.001**	0	**<0.001**
Mid-adulthood only	**0.27 (0.10, 0.44)**		**0.26 (0.09, 0.43)**	
Later adulthood only	0.06 (−0.06, 0.19)		0.06 (−0.07, 0.18)	
Persistent	**0.29 (0.16, 0.42)**		**0.27 (0.13, 0.40)**	

SM: spine mode.

*Mid-adulthood only: back pain reported at age 36 and/or 43 years only; Later adulthood only: back reported at age 53 and/or 60–64 years only; Persistent: Back reported in both mid and later adulthood.

^†^Combined analyses presented except for mode 6 which was sex-stratified, sex interaction, p = 0.048 (unadjusted model); ^¶^adjusted for sex, current BMI, spine aBMD, own occupational class, physical activity, GHQ-28 and number of children.

## Discussion

In a large nationally representative population-based sample of adults aged 60–64 years, higher cumulative exposure to back pain across adulthood was associated with wedge-shaped L4-5 disc (lower SM4 scores) and smaller disc spaces (higher SM8 scores) in both sexes. In addition, back pain reports at one or two ages in adulthood were associated with smaller L4-5 disc space (lower SM6 scores) in men only. When we investigated back pain reports during different periods of adulthood, we found that back pain reports in later adulthood (at ages 53 and/or 60–64 years) were associated with lower SM6 scores (men only) whereas back pain reports in mid-life (at ages 36 and/or 43 years) and across adulthood (mid and later adulthood) were associated with higher SM8 scores. We found no associations between back pain and overall curviness (SM1) or uneven (or snaking) spinal curvatures (SM2).

To the best of our knowledge this is the first study to examine the relationship between back pain at multiple time points across adulthood and spinal shape in early old-age, characterised using statistical shape modelling (SSM). In this study, those who reported back pain at a greater number of ages across adulthood tended to have marginally smaller disc spaces, relative to vertebral heights, at the second to fourth lumbar levels (higher SM8 scores). This association was particularly evident among those who reported early and persistent back pain. In their cross-sectional study, Menezes-Reis *et al*. found a higher frequency of degenerated discs at L4-L5 among 20–40 year old asymptomatic individuals but only in those with subtype II sagittal alignment (flatter lumbar spine)^16^, as described by Roussouly classifications^13^. Although the association between lumbar disc disease (LDD) and low back pain is widely reported to be weak (reviewed in^24^), in a separate study using a different SSM we found that asymptomatic subjects with total scores for LDD of greater than 5, using modified Pfirrmann grading, were also more likely to have smaller L4-S1 disc spaces^26^.

We also found sex differences between back pain and smaller L4-5 disc space (lower SM6 scores). In men, associations were found among those who reported back pain in later adulthood but not among those who reported pain in mid-adulthood only or who had persistent back pain. No associations were found in women.

Our finding of a linear association between cumulative reports of back pain across adulthood and larger relative a-p vertebral diameters (lower SM3 scores) was fully attenuated after adjustment for covariates. This was mainly explained by the adjustment for current BMI. In previous NSHD analyses^27^, onset of overweight at age 36 was also associated with larger relative anterior-posterior (a-p) vertebral diameters in both sexes but this association was fully attenuated after adjusting for current BMI, suggesting that the observed associations may be explained by tracking of BMI across adulthood.

Similarly, the weak linear relationship between cumulative reports of back pain across adulthood and wedge-shaped L4-5 disc (lower SM4 scores) was fully attenuated after adjustment for covariates. However, unlike for SM3, adjustment for leisure time physical activity, higher levels of which were independently associated with lower SM4 scores, had greater effect than adjustment for BMI suggesting a potentially different pathway of association for these two modes.

We found no association between back pain and overall curviness (SM1) or uneven (or snaking) spinal curvatures (SM2). This is consistent with most existing literature reviews; these have reported no evidence of association between sagittal spinal curves (assessed using different measurement methods and position and at a varied number of vertebrae) and back pain^22,23^. One study has reported that individuals with Roussouly subtype II sagittal alignment (flat lordosis) had a higher frequency of degenerated discs at L4-L5 than those with subtype IV (long and curved lumbar spine), but found no significant differences in disc degeneration at other disc levels or with other shapes^16^. However, this sampled asymptomatic individuals and the relationship with low back pain is unclear. In a study of Modic changes identified using MRI among symptomatic patients with low back pain, 53% showed signs of loss of lumbar lordosis^28^; however, this study was limited by the lack of asymptomatic controls and by the use of visual examination of MRI scans to determine lordosis. In contrast, an MRI study which compared lumbar spine morphology (characterised using SSM) in 31 asymptomatic adults aged 45 to 70 years, those with Modic changes were significantly more lordotic, and had a more even curvature, than those without Modic changes^26^.

A key strength of our study is the large population-based sample of adults with back pain ascertained at four time-points across adulthood. We also use SSM to quantify spinal morphology. This is not reliant on angles, which are difficult to measure precisely, and it provides a numerical assessment of subtle variations that might otherwise be overlooked. Limitations relate to the imaging methods employed, the definition of low back pain and bias that may have been introduced due to sample restrictions. DXA spinal images were captured at a single time point; therefore, we are unable to distinguish differences in spine shape due to developmental factors from those due to ageing-related degenerative changes. We are also unable to establish temporality in the relationship with back pain and so acknowledge that the observed associations between back pain and spine shape may reflect associations acting in both directions. Although two-dimensional DXA images are commonly used in large studies, it is conceivable that potential errors during positioning such as the tilt of the pelvis may have occurred; therefore images with extreme rotation were excluded from the SSM models. Similarly, images were acquired in a supine position at all CRFs except one where a fixed C-arm in the scanner was used. However, accounting for CRF had negligible effect on our findings. In addition, our binary measures of back pain, which were selected to facilitate longitudinal analyses, do not capture information on pain severity or its impact which may be a limitation given that greater pain intensity or pain-related disability may influence spinal morphology e.g. by affecting movement and posture. Finally, our analyses were restricted to the sample who attended a CRF as this is where DXA scans were undertaken. It is possible that this restriction may have introduced bias as participants who attended a CRF were less likely to be obese and more likely to be in better health than those who were visited at home^29^.

In conclusion, this study found that higher cumulative exposure to back pain across adulthood was associated with wedge-shaped L4–5 disc (lower SM4 scores) and smaller disc spaces (higher SM8 scores) at age 60–64 in both sexes. In addition, reports of back pain in later adulthood (at ages 53 and/or 60–64 years) were associated with smaller L4–5 disc space (lower SM6 scores) in men but not women. These findings provide evidence which may help inform best strategy on the prevention of back pain.

## Methods

### Study sample

The MRC National Survey of Health and Development (NSHD) is a socially stratified population sample of 5362 single, legitimate births that occurred in England, Wales and Scotland in one week of March 1946 and participants have been prospectively followed regularly ever since. Between 2006 and 2010 (at age 60–64 years), eligible study members known to be alive and living in England, Wales and Scotland were invited for an assessment at one of six clinical research facilities (CRF) or to be visited at home by a research nurse. Of the 2856 invited, 2229 were assessed of whom 1690 attended a CRF.

All waves of data collection have complied with ethical standards. Ethical approval for the data collection at age 60–64 was obtained from the Central Manchester Research Ethics Committee (07/H1008/245) and the Scottish A Research Ethics Committee (08/MRE00/12) and written informed consent was obtained from each participant^30^. The study was carried out in accordance with relevant guidelines and regulations.

### Radiological assessment

During the visits to the CRF, dual-energy X-ray absorptiometry (DXA) images of the lumbar spine were acquired using the QDR 4500 Discovery (Hologic Inc, Bedford, MA) according to standard protocols^30^. Images were obtained with the individuals lying in a supine position in all CRFs except one, where they were scanned in lateral decubitus due to a fixed C-arm in the scanner. All scans were centrally analysed and scrutinized by author JEA’s laboratory.

For quality assurance, Hologic Spine Phantoms provided by the scanner manufacturer were scanned every day prior to participant scanning and in accordance with manufacturer’s protocols, and the results were sent to the coordinating centre once a month for scrutiny^31^.

Standard Bone mineral density measures for the spine were derived using the scanner embedded software provided by the manufacturer.

### Statistical shape modelling

Lumbar spine DXA scans were available for 1601 study participants. The Aberdeen team were responsible for image segmentation, point placement was performed by AVP and ambiguous images and statistical outliers were reviewed by the team in line with the approach outlined in Baird *et al*. and Faber *et al*.^32,33^. Prior to the placement two investigators (AVP, FRS) manually marked a random sample of 50 images from the cohort and intra-rater repeatability was measured as 1.4 pixels and inter-rater repeatability as 2.2 pixels^34^. Of the 1601 images, 72 were excluded due to poor quality, scanning artefacts, incomplete images, evidence of axial rotation or metalwork, leaving 1529 images for statistical shape modelling (SSM)^27^.

The SSM modelling strategy and reasons for image exclusion have been described in detail elsewhere^34^. Briefly, an 89-point template was constructed using custom SSM software (Shape, University of Aberdeen) which described spine shape from the tenth thoracic (T10) to fifth lumbar (L5) vertebrae, marking the vertebral body outlines.

Point outlines in each image were scaled, rotated and translated (Procrustes transformation) to normalise the scale, thus removing size differences. Finally principal components analysis identified orthogonal modes of variation in spine shape; each mode describing a percentage of the total variation in shape (in descending order from mode 1). Each mode of variation has a mean of zero and a score in units of standard deviation. The variance was plotted against each mode in a scree plot^34^ and from this eight modes of variance were selected for analysis; together these modes explained 84.9% of overall shape variance with the largest mode SM1 accounting for 53.0% and the smallest, SM8, 1.2% (Fig. 1b).

### Assessment of back pain

Data on back pain were obtained at nurse interviews during home visits at ages 36, 43, 53 and 60–64 years. At these ages participants were asked whether they had sciatica, lumbago, or recurring/severe backache all or most of the time (ever at ages 36 and 43 years and in the previous 12 months at ages 53 and 60–64 years). We then considered the long-term course of back pain^24^ by including participants with back pain data at three or more time points across adulthood in the analyses.

A variable indicating cumulative exposure to back pain was derived by counting the number of ages at which back pain was reported. The derived score (range, 0 (no reports of back pain) to 4 (back pain reported at all 4 ages) was categorised into 4 groups (0, 1, 2, 3–4).

To distinguish between individuals who experienced back pain in different phases of adulthood, so that the importance of timing of exposure could be assessed, a variable was derived with 4 categories as follows: (i) no back pain (i.e. those who did not report back pain at any visit or at 3 visits if data at one visit were missing); (ii) back pain in mid-adulthood only (i.e. those who reported back pain at ages 36 and/or 43 years but not at later ages); (iii) back pain in later adulthood only (i.e. those who reported back pain at ages 53 and/or 60–64 years but not at earlier ages); (iv) back pain across adult life (i.e. those who reported back pain in both mid (36 and/or 43 years) and later adulthood (53 and/or 60–64 years)).

### Covariates

Weight (kg) and height (m) were measured at age 60–64 years by a trained nurse and body mass index (BMI) was calculated as weight (kg)/(height (m))^2^. Lumbar spine (L1-L4) areal bone mineral density (aBMD) was also obtained at this age^27^.

Own occupation at age 53 years was categorised according to the Registrar General’s social classification (ONS, 1990) into three groups: high (I or II); middle (IIINM or IIIM); low (IV or V).

Symptoms of anxiety and depression were assessed at age 60–64 years using the 28-item General Health Questionnaire (GHQ-28). Each item was coded using the General Scoring Method then summed with a threshold for caseness of 5 or more selected^35^.

Self-reported level of participation in sports, vigorous leisure activities or exercise was assessed at age 60–64 years and grouped as inactive, moderately active (1–4 times/month) or regularly active (≥5 times/month).

Information on parity was obtained from study members prospective reports across adulthood updated to age 53 years and grouped as 0; 1; 2; ≥3 children.

### Statistical analysis

Using linear regression models, we first examined the associations between cumulative exposure to back pain across adulthood (no reports of back pain, 1, 2, 3 or more back pain reports) and each spine shape mode (SM1–8) at age 60–64 (aim 1). We then investigated whether timing of back pain (no reports of back pain, early-adulthood, mid-adulthood and persistent back pain) was associated with spine shape (aim 2). Models were first adjusted for sex (with formal tests of sex interaction undertaken and subsequent models sex-stratified where evidence of this was found) and then for current BMI, spine BMD, own occupational class, physical activity, GHQ-28 and parity.

All models included participants with complete data on back pain data (at least 3 visits), spine mode scores and covariates (N = 1405).

All analyses were conducted using STATA v14.1. P-values (two-tailed) are reported.

## Data Availability

Data are available on request to the NSHD Data Sharing Committee. NSHD data sharing policies and processes meet the requirements and expectations of the UK Medical Research Council (MRC) policy on sharing of data from population and patient cohorts: https://www.mrc.ac.uk/publications/browse/mrc-policy-and-guidance-on-sharing-of-research-data-from-population-and-patient-studies/. Data requests should be submitted to mrclha.swiftinfo@ucl.ac.uk; further details can be found at http://www.nshd.mrc.ac.uk/data.aspx. These policies and processes are in place to ensure that the use of data from this national birth cohort study is within the bounds of consent given previously by study members, complies with MRC guidance on ethics and research governance, and meets rigorous MRC data security standards.
